# Enhancing Stiffness and Oil Resistance of Fluorosilicone Rubber Composites through Untreated Cellulose Reinforcement

**DOI:** 10.3390/polym15234489

**Published:** 2023-11-22

**Authors:** Ye-Won Park, Jeong-Hwan Yoon, Kyoung-Ho Shin, Yeon-Jee Cho, Ju-Ho Yun, Won-Hee Han, Min-Hyuk Hong, Dong-Gug Kang, Hye-Young Kim

**Affiliations:** 1 Chemical Materials R&D Department, Chassis & Material Research Laboratory, Korea Automotive Technology Institute, 303 Pungse-ro, Pungse-myeon, Dongnam-gu, Cheonan-si 31214, Chungnam, Republic of Korea; ywpark1@katech.re.kr (Y.-W.P.); khshin@katech.re.kr (K.-H.S.); yjcho@katech.re.kr (Y.-J.C.); jhyun@katech.re.kr (J.-H.Y.); 2 R&D Centre, Lion Advanced Materials Inc., 87 Beotkkot-gil, Daedeok-gu, Daejeon 34342, Republic of Korea; 3 Research & Development Institute, Pyung Hwa Oil Seal Industry Co., Ltd., 42 Nongongjungang-ro 51-gil, Nongong-eup, Dalseong-gun, Daegu 42982, Republic of Korea

**Keywords:** fluorosilicone rubber, untreated cellulose, liquid-assisted mixing, oil resistance, mechanical properties, stress relaxation

## Abstract

Fluorosilicone rubber, essential in automotive and aerospace owing to its excellent chemical resistance, plays a pivotal role in sealing technology, addressing the industry’s evolving demands. This study explores the preparation and properties of fibrillated cellulose-reinforced fluorosilicone rubber composites to enhance their stiffness and oil resistance. Fibrillated cellulose sourced as a wet cake and subjected to processing and modification is incorporated into a fluorosilicone rubber matrix. The resulting composites are analysed by tensile and compression tests, along with compressive stress-relaxation testing in air and in an oil-immersed environment. The findings demonstrate significant improvements in the mechanical properties, including an increased Young’s modulus and elongation at break, whereas the tensile strength remained uncompromised throughout the testing procedures. Morphological analysis of the fracture surfaces revealed a remarkable interfacial affinity between the fibrillated cellulose and rubber matrix, which was attributed in part to the modified fatty acids and inorganic nanoparticles. The presence of fibrillated cellulose enhanced the stress-relaxation characteristics under oil-immersion conditions. These results contribute to the domain of advanced elastomer materials, with potential for applications requiring enhanced mechanical properties and superior oil resistance.

## 1. Introduction

Fluorosilicone rubber composites are indispensable materials across diverse industries owing to their extraordinary resilience to challenging environments, including extreme temperatures and corrosive substances, which has rendered these materials invaluable in applications spanning the automotive, aerospace, and oil and gas sectors [1,2,3,4,5]. The pursuit of composites with superior elastomeric properties remains a high priority in the dynamic realms of materials science and engineering.

The performance of rubber matrices is conventionally improved by the incorporation of reinforcing agents into rubber matrices [6,7,8]. Carbon black (CB), a common reinforcing agent in the rubber industry, has long been the standard for enhancing the properties of rubber; however, the provenance of CB from petroleum sources raises environmental concerns, underlining the urgent need to identify more sustainable alternatives [9,10,11,12]. Natural cellulose is a robust candidate for improving the performance of rubber composites. Cellulose is abundant, renewable, lightweight, and eco-friendly. This unique combination of properties makes it an excellent potential reinforcing agent to improve the mechanical properties of natural rubber, silicone rubber, and styrene-butadiene rubber [13,14,15,16,17,18,19,20,21,22,23,24]. Surface treatment has conventionally been an essential step in augmenting the affinity of cellulose for a rubber matrix [25,26]; however, surface treatment often offsets some of the inherent benefits of cellulose incorporation. Therefore, the utilisation of untreated cellulose to improve rubber performance and explore novel applications requires attention.

Liquid-assisted extrusion has emerged as a promising technology for enhancing cellulose dispersibility within polymer matrices and has attracted significant research attention, particularly with water as a facilitator. The liquid in this process acts as a lubricant, facilitating dispersion and preventing agglomeration. Although this approach was initially applied to biopolymer-based composites, its recent extension to polyolefins has further underscored its versatility [27].

The core premise of the present study is that the dispersal of fibrillated cellulose within a fluorosilicone rubber matrix induces a synergetic improvement in the properties of the composite. Our study examines how the distribution of fibrillated cellulose influences the mechanical properties and oil resistance of the composite. A comprehensive analysis was conducted that included tensile, compression, and compressive stress-relaxation (CSR) tests, all performed under conditions replicating automotive sealing environments. In a broader context, this study aimed to decisively bridge the gap between conventional rubber composites and advanced elastomeric materials. The study provides insights that can reshape the landscape of material design. The impact of the dispersal of fibrillated cellulose on the properties of fluorosilicone rubber is significant, not only from an academic perspective, but also for its potential to catalyse the development of materials with augmented mechanical robustness and heightened resistance to oil penetration. Furthermore, this study adds to the findings of numerous studies exploring the synergistic potential of natural fillers in polymer matrices, further illuminating the association between structural and mechanistic properties.

## 2. Materials and Methods

### 2.1. Preparation of Fibrillated Cellulose

Fibrillated cellulose, derived from wood pulp, was sourced as wet cake from Moorim P&P Inc. (average diameter ≤200 nm, aspect ratio >50, 85.0 wt% water, Ulsan, Republic of Korea). The mechanical wet exfoliation technique was employed in the manufacturing process. Prior to concentration, the water content of the wet cake ranged from 97.8 to 99.0 wt%.

**Figure 1:**
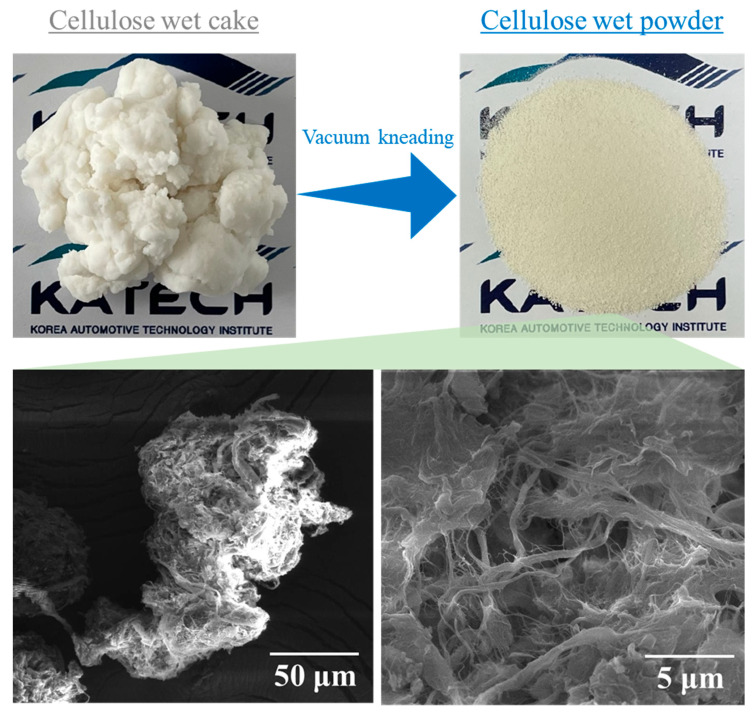
Photographs and SEM images of wet fibrillated cellulose powder.

The wet cake was modified to further facilitate processing and dispersal within the composite. The wet cake was blended to incorporate 5 wt% of a modified fatty acid, designated as SEO-5, with a modification ratio of 5.0%. SEO-5 has a dropping point of 85 °C and a specific gravity of 1.0 g/cm³. Additionally, 10 wt% of hydrophilic inorganic nanoparticles ranging from 20–30 nm in diameter and with a density of 5.6 g/cm³ were incorporated into the material through a vacuum kneading process. (These materials were synthesised using proprietary technology provided by Lion Advanced Materials Inc. (Daejeon, Republic of Korea) and are available commercially. Further details cannot be provided here owing to confidentiality constraints). Subsequently, the solid content of the resulting wet powder was adjusted to maintain a uniform consistency, targeting a value of 50 ± 2 wt%. Figure 1 shows a photograph of the wet powder, accompanied by an SEM image obtained after freeze-drying, further elucidating the structural characteristics of the fibrillated cellulose used. The additives are intended solely as processing aids for pulverisation and do not participate in or otherwise influence the reactions; however, they can function as dispersing agents that are distributed primarily on the cellulose surface and thus prevent cohesion between cellulose molecules.

### 2.2. Sample Preparation

The fluorosilicone polymer, denoted FE-271-U, with a fluorine content of 0.8%, and the peroxide-based cross-linking agent, C-8A, were obtained from ShinEtsu (Tokyo, Japan). To achieve optimal cross-linking, the cross-linking agent was introduced at the recommended concentration of 1 part per hundred resins (phr), following the manufacturer’s guidelines. A preliminary batch of the polymer (1 kg) was premixed with the cross-linking agent using an 8-inch open roll machine, and the fibrillated cellulose was subsequently incorporated into the mixture at concentrations of 1.25, 2.50, and 3.75 phr, as all the moisture within the fibrillated cellulose wet powder was removed during this process. The samples were labelled based on their respective final concentrations. The mixing process lasted 15 min, during which time the silicone-specific adhesiveness progressively reduced with increasing cellulose content. A critical point was reached during the mixing phase when the cellulose concentration reached 5.00 phr and delamination occurred during the roll milling process. Consequently, the corresponding sample was excluded from the subsequent experiments.

Test samples were fabricated from the polymer mixtures using a plate-type vulcaniser. The vulcanisation apparatus featured an outlet for discharging water vapour and ensured a constant pressure throughout the vulcanisation process. Vulcanisation was performed at 180 °C for 6 min, maintaining a pressure of 10 MPa.

### 2.3. Testing Procedures

#### 2.3.1. Tensile Testing

Tensile testing was performed using ISO 37 Type 1 dumbbell-shaped test specimens precision-cut from the moulded samples, each having a uniform thickness of 2 mm and horizontal and vertical lengths of 100 mm [28]. Tensile tests were conducted using a universal testing machine (AG-X plus, Shimadzu Co., Kyoto, Japan) equipped with a 500 N load cell. A pneumatic grip mechanism was employed to firmly secure the test specimen. After securing the test specimen, an initial preload of 0.5 N was applied to establish the zero-point reference. Given the considerable elongation at the yield points of the test specimens, a testing speed of 500 mm/min was used to ensure reliable results. To ensure a comprehensive assessment of the mechanical behaviour of the prepared samples, cyclic tensile tests were performed to analyse their hysteresis characteristics. Each test comprised ten cycles, with each cycle involving stretching the test specimen by 10% of the gauge distance and subsequently restoring it until the load reached 0. The resultant stress and hysteresis loss were recorded during each cycle.

#### 2.3.2. Compression Testing

In the compression testing phase, a universal testing machine equipped with a 5 kN load cell was employed to accommodate the higher load levels experienced during compression testing (compared to tensile testing). The specimens used for compression testing were fabricated in accordance with ISO 7743 Type A [29]. The specimens were cylindrical disks, each with a diameter of 29 mm and a length of 12.5 mm. To minimise the potential influence of irregular slippage during testing, a thin layer of silicone oil was applied to the upper and lower surfaces of each test specimen. The compression process was executed at a controlled speed of 10 mm/min, during which the specimen was compressed to 25% of its original height from a preload condition of 0.5 N and then restored to its initial state. This compression and restoration cycle was repeated four times, and the results from the fourth cycle were used in subsequent analyses.

#### 2.3.3. Compressive Stress-Relaxation Testing

The CSR test was conducted in accordance with ISO3384-1 A using a dedicated relaxation system (Stress Relaxation Test System; Elastocon AB Co., Brämhult, Sweden) designed for continuous rubber testing [30]. Cylindrical test specimens were prepared, each with a diameter of 13 mm and a thickness of 6.3 mm. Each test specimen was compressed to 25% of its initial thickness and maintained in this compressed state for the entire duration of the CSR test, which spanned 1008 h and was conducted at 150 °C. To facilitate the CSR testing in an oil-immersed environment, a stress-relaxation rig (EB 02, Elastocon AB Co., Brämhult, Sweden) equipped with mineral oil (15W40; Shell Helix Co., Brämhult, Sweden) and a liquid container for the necessary immersion were employed. During compression, the test specimen was securely positioned within the stress-relaxation rig and submerged in mineral oil in the liquid container. The entire CSR test process, including control, monitoring, and data recording, was managed using a dedicated testing software (ED 04, Elastocon AB Co., Brämhult, Sweden).

## 3. Results and Discussion

### 3.1. Enhancement of Mechanical Properties 

#### 3.1.1. Tensile Properties

**Figure 2:**
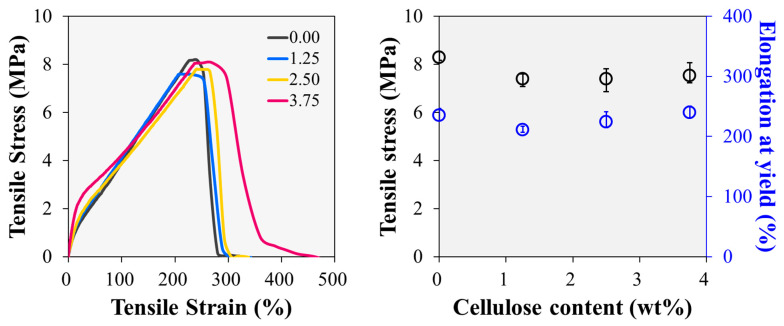
Tensile properties of fluorosilicone rubber samples with various fibrillated cellulose contents.

Figure 2 shows the stress–strain curves obtained from the tensile tests of the fluorosilicone rubber samples with various cellulose contents. Distinct enhancements in the Young’s modulus and elongation at break are observed with increasing cellulose content. A sharp increase is observed in the sample containing 3.75 phr of cellulose, compared to the 2.50 phr sample, which reflects the pivotal advantage associated with the liquid-assisted extrusion process employed in this study [31,32,33]. This method, which involves mixing cellulose and polymers in the presence of water, enhances the dispersion of fibrillated cellulose and the formation of a percolating network [34,35]. The observed phenomenon distinguishes the effect of cellulose observed in this study from the traditional reinforcing effects attributed to common fillers, which typically manifest as increased stiffness and reduced elongation. In contrast to the increases in Young’s modulus and elongation at break, the maximum strength of the fluorosilicone rubber sample exhibited a slight reduction in the presence of cellulose. This reduction was attributed to the absence of robust interfacial bonding between the cellulose filler and the polymer matrix. As the sample deformed at high elongation, physical separation occurred at the filler–matrix interface, weakening the interactions among the dispersed cellulose particles. Despite these responses, the highest tensile strength of the 3.75 phr sample was 8.1 MPa, which is similar to the tensile strength of the 0 phr sample (8.2 MPa).

**Figure 3:**
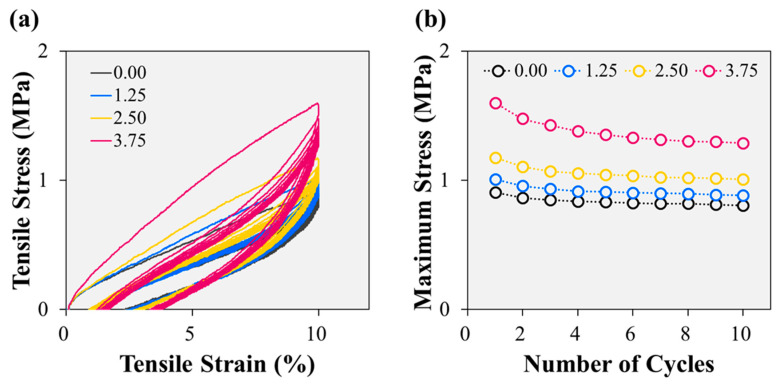
Cyclic tensile tests of fluorosilicone rubber samples with various fibrillated cellulose contents: (**a**) stress–strain curves, and (**b**) maximum stress vs. number of cycles.

The behaviour of the fluorosilicone rubber samples under cyclic loading conditions was also investigated (Figure 3a). The observed hysteresis loop encircled by the loading and unloading curves revealed the presence of substantial residual strain [36]. The stress and hysteresis loops demonstrated a consistent trend characterised by a rapid reduction from the first cycle to the second cycle, followed by a more gradual decline. This phenomenon was attributed to the Mullins effect, which becomes increasingly pronounced with increasing filler content [37]. This trend implies the formation of a more extensive physical network within the polymer matrix, owing to the elevated fibrillated cellulose content [38]. This enhanced network formation is confirmed by the change in the maximum tensile stress values extracted from each cycle, shown in Figure 3b. Notwithstanding the influence of the Mullins effect, the inclusion of fibrillated cellulose enables fluorosilicone rubber to maintain greater stress under repeated deformations. In the tenth cycle, the maximum stress of the 3.75 phr sample (1.29 MPa) was approximately 59% higher than that of the 0.00 phr sample (0.81 MPa). Concurrently, the 1.25 phr and 2.50 phr samples exhibited maximum stresses of 0.88 MPa and 1.01 MPa, respectively. As the cellulose content increased, the maximum stress in the tenth cycle increased; however, the difference is smaller than observed in the first cycle owing to the Mullins effect.

#### 3.1.2. Compression Strength

**Figure 4:**
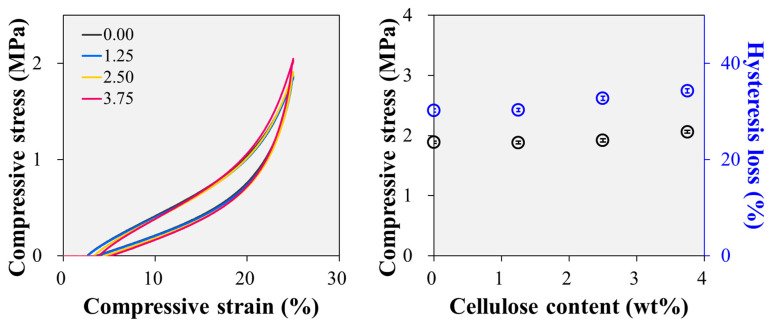
Compressive stress and hysteresis loss of the samples up to 25% compression.

The compression testing results presented in Figure 4 offer valuable insights into the mechanical behaviour of the fluorosilicone rubber samples. The stress–strain curves reveal the presence of significant residual strain, the magnitude of which exhibits a significant correlation with the cellulose content. Intriguingly, an increase in the cellulose content is associated with a steeper slope in the stress–strain curve. Consequently, the stress at 25% compressive strain exhibits a remarkable variation, with the sample containing 3.75 phr cellulose exhibiting a stress of 2.04 MPa, approximately 9% higher than that of the sample devoid of cellulose (1.87 MPa). These findings have important implications for enhancing the performance of fluorosilicone rubbers in practical applications. As an example, we consider the use of fluorosilicone rubber seals in automotive vacuum pumps, where a compression of approximately 25% is essential to ensure effective sealing. In such commercialised and operational scenarios, materials displaying higher stress levels can accommodate more robust sealing forces, demonstrating the advantages of cellulose-reinforced fluorosilicone rubber in practical applications.

### 3.2. Morphological Analysis of Fracture Surfaces

**Figure 5:**
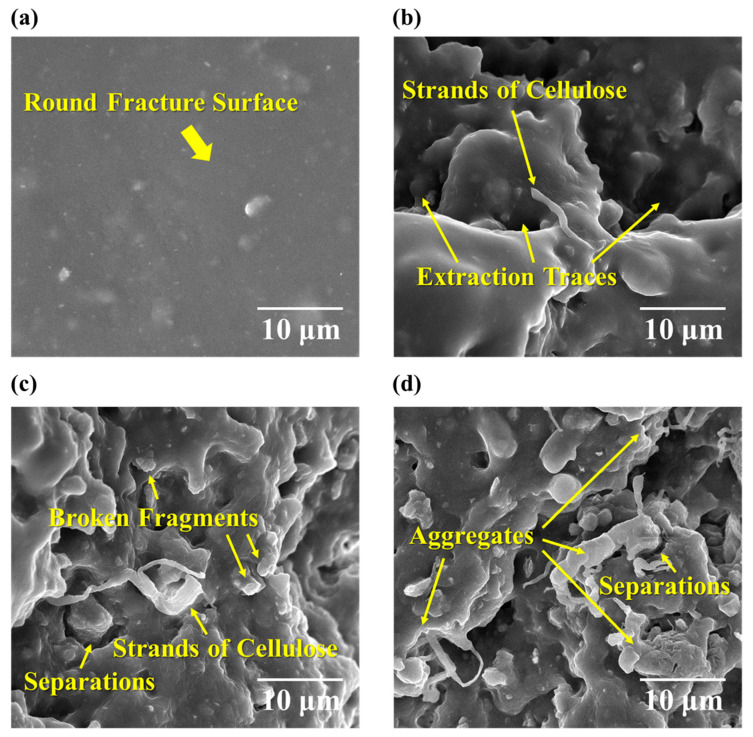
SEM images of tensile fracture surfaces in samples with various fibrillated cellulose contents: (**a**) 0.00 phr, (**b**) 1.25 phr, (**c**) 2.50 phr, and (**d**) 3.75 phr.

To reveal the dispersed structure of the fibrillated cellulose within the fluorosilicone rubber matrix and investigate the morphological aspects of the reinforcement mechanism, the fracture surfaces of the samples subjected to tensile testing were examined using microscopic techniques. Figure 5 shows the SEM images of the tensile fracture surfaces of each sample. The cellulose-free sample exhibits a round, smooth fracture surface (Figure 5a. In contrast, the samples containing cellulose (Figure 5b–d) exhibit a considerably different, rough, and fractured morphology. These images reveal the presence of matrix aggregates formed by the entanglement of fibrillated cellulose, as well as individual cellulose strands dispersed throughout the fracture surface. The cellulose strands are consistently enveloped by the surrounding fluorosilicone rubber matrix, suggesting an interfacial affinity between the cellulose and polymer matrix. This interfacial affinity may be attributed to the presence of modified fatty acids and inorganic nanoparticles. The additives, which are used to pulverise the cellulose wet cake and hinder cohesion among cellulose molecules, likely play a significant role in enhancing the interactions between cellulose and the polymer matrix. During the mixing process, the interactions between cellulose and the polymer matrix improve as the number of incohesive cellulose strands increases. These strands form more cohesive structures with the rubber, as evidenced by the increased number of aggregates (Figure 5d). Although these aggregates are enveloped by fluorosilicone rubber, they are physically separated from the surrounding polymer matrix, which indicates that cohesive forces within the fibrillated cellulose aggregates are stronger than the interactions between the aggregates and the fluorosilicone rubber matrix, as observed on the fracture surfaces. This explains the tensile test results presented in Figure 2. The matrix encompassing the surfaces of these aggregates or cellulose strands resists deformation with increasing force, thereby increasing Young’s modulus. In contrast, the slope of the stress–strain curve decreases as the cellulose content increases, specifically in the deformation region spanning from the elastic linear phase to the yield point. This decline may be attributed to the sequential initiation of the separation of areas with relatively weak interfacial adhesion. Consequently, the separation region expanded as the cellulose content increased. Nevertheless, the aggregates, characterised by their heightened cohesion, resist deformation more effectively than the surrounding matrix. This explains the increase in strain from the yield point to the fracture point.

### 3.3. Oil Resistance

**Figure 6:**
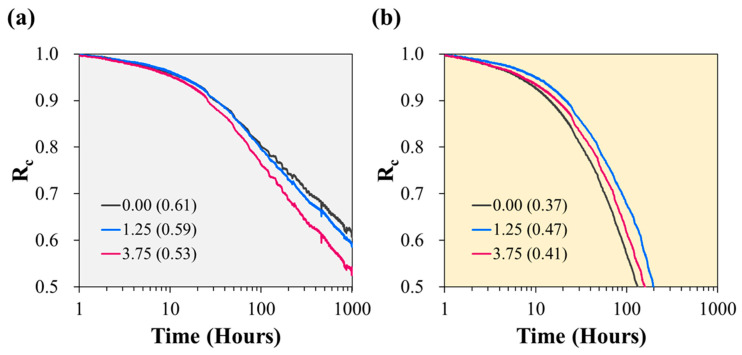
Change in R_c_ of samples with different cellulose contents over time: (**a**) in an air environment, and (**b**) in an oil-immersed environment.

The oil resistance of the samples was evaluated using CSR testing, considering practical field conditions similar to those of oil seal products. Tests were conducted on samples with cellulose contents of 0.00, 1.25, and 3.75 phr to evaluate the impact of cellulose on oil resistance. Six test cells were prepared and simultaneously assessed under two distinct conditions: air immersion and oil immersion, each conducted at 150 °C. Figure 6 shows the normalised continuous stress relaxation, denoted R_c_, defined as the ratio of the continuously measured force to the initial force applied during compression. The behaviour of R_c_ in the air is characterised by the sample without cellulose displaying a gentle slope (Figure 6a), indicating excellent stress-relaxation characteristics [39]. In contrast, the 3.75 phr sample, which had a high cellulose content, exhibits poor stress-relaxation characteristics, manifesting the steepest slope. This outcome contrasts with the typical behaviour of rubber elastomers containing conventional fillers. Conventionally, short fibres, such as glass or carbon, typically impede chain mobility owing to their interfacial interactions with the polymer matrix, which reduces the stress-relaxation rate [40,41,42,43,44,45,46,47,48,49,50,51]. This unexpected result can be attributed to the unique properties of the fluorosilicone rubber samples in which the fibrillated cellulose was dispersed. Fibrillated cellulose, characterised by its substantial aspect ratio and flexibility, may reduce the impact of filler–polymer interactions. Under strain, the flexible nature of the cellulose strands allows them to respond harmoniously to the polymer matrix without substantial resistance to deformation. In particular, fibrillated cellulose primarily forms entangled aggregates with the polymer, influencing the response to deformation, which may be predominantly derived from interactions between the polymer surrounding the cellulose aggregates and the surrounding matrix, rather than from filler–filler or filler–polymer interactions. Separation is assumed in areas with relatively weak interfacial adhesion, leading to the gradual expansion of these separation zones and ultimately promoting stress loss. After a test lasting 1008 h, the R_c_ of the 3.75 phr sample was 0.53, 0.08 units lower than that of the 0.00 phr sample (0.61).

In contrast, the behaviour of R_c_ exhibits a notably different trend in the oil-immersed environment (Figure 6b). Stress relaxation in the oil-immersed conditions occurred significantly more swiftly than in the air environment. This rapid stress relaxation can be attributed to swelling, which reduces the rigidity of the rubber, independent of the stress-softening or stress-relaxation phenomena [52]. Consequently, the R_c_ values of all samples had fallen below 0.5 by 200 h; thus, the test was concluded after 216 h, and the R_c_ values at that point were compared. The 0.00 phr sample, which exhibited the highest R_c_ in the air environment, exhibited the lowest R_c_ (0.37) in the oil-immersed environment, whereas the 1.25 phr sample and the 3.75 phr sample exhibited R_c_ values of 0.47 and 0.41, respectively, with the 1.25 phr sample displaying the most favourable characteristics. The R_c_ values of both of these samples were 0.12 units lower than those measured during the 1008 h test in air. This emphasises that the reduced characteristics of the 3.75 phr sample in the oil-immersed environment can be primarily attributed to the initial low CSR characteristics of the sample.

### 3.4. Mechanistic Insights for Probing Mechanisms

**Figure 7:**
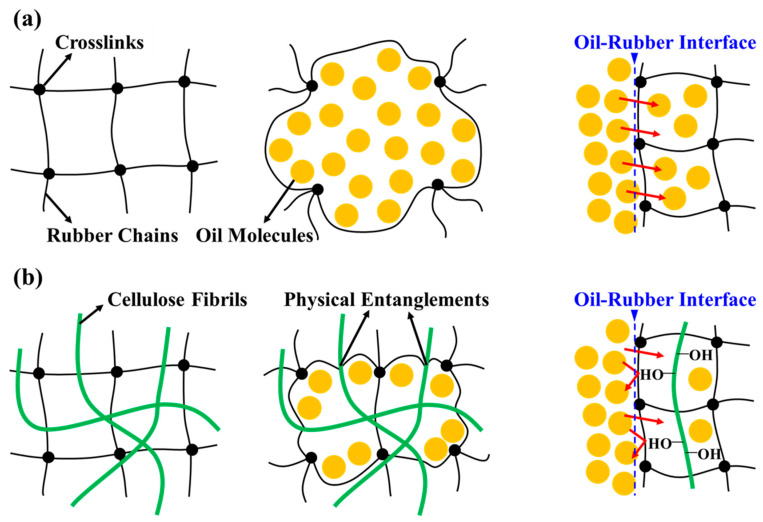
Absorption of oil molecules within the crosslinked network of fluorosilicone rubber: (**a**) 0.00 phr sample without cellulose, and (**b**) other samples containing cellulose.

We experimentally validated that the properties of fluorosilicone rubber were substantially altered by the dispersal of fibrillated cellulose. The effective dispersal of robustly hydrophilic cellulose within the polymer matrix imparted distinct characteristics to the composites. In particular, the surface energy and hydrophobicity of the resulting composites differed from those of the pristine polymer. Empirical studies have revealed an increase in the contact angle of water droplets on the surface of the composite material owing to the presence of microcrystalline cellulose integrated into the silicone rubber [53]. A parallel assessment of the swelling behaviour conclusively established that swelling was intimately linked to interactions within the rubber–solvent matrix system [54]. Samples characterised by heightened swelling levels exhibited a lower stress-to-strain ratio, indicating more robust rubber-solvent interactions. Figure 7 shows the absorption of oil molecules within the crosslinked network of fluorosilicone rubber. The absorption of oil molecules by a 0.00 phr sample without cellulose (Figure 7a) unfolds relatively seamlessly, with the dominant factor being the rubber–solvent interaction. Figure 7b shows the oil absorption of samples containing cellulose. The presence of dispersed fibrillated cellulose exerts an additional influence on the swelling properties of the composite. The physical entanglement between the cellulose and rubber chains in aggregates that were intricately entangled with cellulose hindered the absorption of oil molecules. Furthermore, the absorption of oil molecules at the interface of the sample surface with oil was further diminished owing to the heightened hydrophilicity of the composite. In summary, the effect of the dispersal of fibrillated cellulose may be ascribed to both the inherent attributes of cellulose and the intricately entangled dispersion structure between cellulose and the rubber matrix. Consequently, the dispersal of cellulose improved the rigidity of the fluorosilicone rubber and its enhanced resistance to oil. This experimental evidence elucidates the profound impact of the dispersion of fibrillated cellulose on the performance of the composite.

## 4. Conclusions

This study investigated the preparation and properties of fibrillated cellulose-reinforced fluorosilicone rubber composites and the enhancement of the composites’ stiffness and oil resistance, with a particular focus on the impact of well-dispersed fibrillated cellulose within a fluorosilicone rubber matrix. Fibrillated cellulose, which was procured as a wet cake, was processed and modified with the inclusion of modified fatty acids and hydrophilic inorganic nanoparticles. The resulting composites were analysed by tensile and compression tests, which were conducted under standard conditions, whereas CSR testing was performed in both air- and oil-immersed environments.

Our findings reveal significant enhancements in the mechanical properties of fluorosilicone rubber composites owing to the presence of fibrillated cellulose. Specifically, Young’s modulus and the elongation at break both increased, demonstrating the efficacy of fibrillated cellulose in reinforcing the material. Interestingly, the presence of cellulose did not compromise the overall tensile strength, indicating the potential of fibrillated cellulose as a filler in rubber composites. A morphological analysis of the fracture surfaces elucidated the mechanisms underlying the observed improvements in performance. SEM images demonstrated that the fibrillated cellulose strands were enveloped by the surrounding fluorosilicone rubber matrix, confirming the interfacial affinity between the cellulose and the polymer matrix, which was attributed to the influence of modified fatty acids and inorganic nanoparticles that served as effective dispersing agents, thereby enhancing cohesion. In the oil-resistant domain, the addition of fibrillated cellulose had contrasting effects under different conditions. CSR testing indicated that the presence of cellulose enhanced stress-relaxation characteristics in an oil-immersed environment.

The results confirm the significant impact of the dispersal of fibrillated cellulose on the performance of fluorosilicone rubber composites and highlight the potential of these composites for applications requiring enhanced mechanical properties and oil resistance. The insights obtained in this study contribute to the body of knowledge in the field of advanced elastomer materials and offer a promising avenue for future research and practical applications.

## 5. Patents

A patent application was filed at the Korea Automotive Technology Institute (KATECH).

## Data Availability

The data presented in this study are available on request from the corresponding author.
